# Robust Control for the Detection Threshold of CFAR Process in Cluttered Environments

**DOI:** 10.3390/s20143904

**Published:** 2020-07-13

**Authors:** Jeong Hoon Shin, Youngjin Choi

**Affiliations:** 1 1st Research and Development Institute, Agency for Defense Development, Daejeon 34060, Korea; jhshin75@add.re.kr; 2 Department of Electronic Systems Engineering, Hanyang University, Ansan 15588, Korea

**Keywords:** CFAR process, robust control, clutter measurement density, probability of target existence

## Abstract

The constant false alarm rate (CFAR) process is essential for target detection in radar systems. Although the detection performance of the CFAR process is normally guaranteed in noise-limited environments, it may be dramatically degraded in clutter-limited environments since the probabilistic characteristics for clutter are unknown. Therefore, sophisticated CFAR processes that suppress the effect of clutter can be used in actual applications. However, these methods have the fundamental limitation of detection performance because there is no feedback structure in terms of the probability of false alarm for determining the detection threshold. This paper presents a robust control scheme for adjusting the detection threshold of the CFAR process while estimating the clutter measurement density (CMD) that uses only the measurement sets over a finite time interval in order to adapt to time-varying cluttered environments, and the probability of target existence with finite measurement sets required for estimating CMD is derived. The improved performance of the proposed method was verified by simulation experiments for heterogeneous situations.

## 1. Introduction

The detection process in a radar system is a crucial part of examining radar signal returns and determining whether a target is present within a region of interest (ROI). While a target detection can be simply declared if the amplitude of a received signal is higher than a fixed threshold level, both a target signal and background interference, such as a clutter signal, originally have random amplitude variation, and thus the background interference may exceed the threshold or the target signal may fail to surpass it. The detection performance of a radar system is usually characterized by the probability that a target signal is detected, the probability of detection ($P_{D}$), and the probability that a detection is declared when no target is present, the probability of false alarm ($P_{\mathrm{FA}}$) [1,2].

In general, a radar system is designed to achieve and retain a desired probability of false alarm ($P_{\mathrm{FA},D}$) in the detection process because overall system performance can be degraded by false alarms that may be considered as the detection results of valid targets [2]. The constant false alarm rate (CFAR) process makes it possible by estimating changes in the representative level for background interference and automatically adjusting a threshold level to maintain $P_{\mathrm{FA},D}$ given that the statistics of background interference are known exactly. However, the form of the probability density function (PDF) for background interference can generally be known, but the parameters related to the PDF cannot be known accurately because these terms are varied temporally or spatially as environmental conditions are changed in time [3]. If the basic CFAR process, such as cell-averaging CFAR (CA-CFAR), is applied in cluttered environments with unknown information for the PDF of clutter signals, $P_{\mathrm{FA}}$ may be increased or $P_{D}$ may be decreased as the CFAR loss caused by the estimation error for background interference is raised. Hence, the detection performance of the basic CFAR process may be seriously degraded in cluttered environments owing to a prior uncertainty for the PDF. The robust CFAR process, such as order statistics CFAR (OS-CFAR), can overcome the vulnerability of the basic CFAR process to some degree by ignoring the effect of clutter for determining the detection threshold. However, the robustness of the CFAR process is acquired at the expense of increased CFAR loss and higher processing load. Moreover, it also requires prior knowledge of background interference to achieve good performance [1]. For the OS-CFAR process, the percentile of the data sorted by amplitude within a CFAR window has to be determined to find the statistic for background interference. The adaptive CFAR process, such as heterogeneous clutter estimating CFAR (HCE-CFAR) and generalized censored mean level detector (GCMLD), can be designed adaptively to determine the design parameters of the CFAR process with information from the measured data, rather than a designer’s assumption for background interference. For example, the HCE-CFAR process tries to find the location of the clutter boundary within a CFAR window and uses only samples associated with the same PDF for estimating the statistic of background interference, and the GCMLD process attempts to seek the number and the location of interfering target signals contained within a CFAR window and discards these components for target masking before estimating the statistic [2]. Furthermore, the refined CFAR process, such as fusing a robust CFAR process with a basic CFAR process for estimating the statistic adaptively [4,5] or seeking the exact model for the PDF of background interference [6], has been researched. However, these CFAR processes fundamentally have the potentiality to increase $P_{\mathrm{FA}}$ in cluttered environments because the methods mainly focus on the $P_{D}$ for a target and have an open loop structure in terms of $P_{\mathrm{FA}}$, i.e., the error of $P_{\mathrm{FA}}$ caused by the model uncertainty for the PDF of background interference does not affect determining the detection threshold.

An approach for adjusting the detection threshold of the CFAR process with the feedback control loop based on the clutter measurement density (CMD) is proposed [7], in which the extremum seeking concept as a nonlinear adaptive controller is introduced since the CFAR process has strongly nonlinear transfer characteristics, and the standard CMD estimator (CMDE) based on the probability of target existence (PTE) is applied for retaining $P_{\mathrm{FA},D}$ in a cluttered environment. The CMD, defined as the number of measurements for clutter signals per unit area, is linked with $P_{\mathrm{FA}}$ and usually applied for calculating the measurement likelihood ratio of the posterior PDF for the tracking filter. The PTE is the probability of whether there exists a target within an ROI based on the acquired measurements with the information of uncertain origin and is usually used as a measure of tracking quality for track initiation, confirmation, and termination in target tracking [8]. Whereas the solution with the extremum seeking controller is devoted to control the CMD given that the input contains only clutter signals, the situation that both target and clutter signals exist within an ROI must be additionally considered for actual applications. Furthermore, the CMDE is the key module for building the CFAR process with a feedback loop structure. In general, the CMD can be simply assumed to be a constant, but it will be better to estimate the CMD adaptively because the CMD has no prior information and can be changed significantly as the conditions of the operating environment are changed [9,10,11]. Hence, various CMDEs have been researched to enhance the tracking accuracy for tracking filters in cluttered environments. Given that clutter signals are uniformly distributed in space and Poisson distributed in time, the heuristic method based on the PTE [8], the conditional mean and the maximum likelihood method based on the notion of target perceivability [12,13] are the representatives of track-oriented estimators with the information of all acquired measurement sets up to the current time. However, the status of target existence and the CMD will be changed continuously in time, especially in a heterogeneous cluttered environment, so it may be difficult to estimate the current information of the PTE and the CMD with all past observations more rapidly and precisely.

This paper suggests an approach for controlling the detection threshold of the CFAR process robustly based on the CMDE with the measurement sets over a finite time interval. Furthermore, the PTE with finite measurement sets is derived for applying the CMDE. A robust control technique can be appropriate to manage the uncertain characteristics for the PDF of clutter signals, and applying finite measurement sets can be helpful for the CFAR process to adapt well to dynamic cluttered environments.

The rest of this paper is organized as follows: the problem statement is defined in Section 2, the proposed robust control scheme is elaborated in Section 3, the simulation experiments are shown in Section 4, followed by the conclusions in Section 5.

## 2. Problem Statement

If some signals are detected in a radar system, radar resources are allocated to other functions consecutively, such as verification, data association, and tracking. False alarms can occur in the detection process owing to the inherent randomness of received signals and a large number of false alarms can overload the radar resources. This may result in abnormal detecting or tracking operation and consequently degrade the performance of the entire radar system. Hence, it is the vital point in a radar system to maintain a false alarm rate at a desired level.

The false alarm rate (FAR) means the number of false alarms per a given time interval and is proportional to $P_{\mathrm{FA}}$ by

(1) $$\begin{array}{c} \mathrm{FAR}=\frac{N_{\mathrm{FA}}}{T_{N}}=\left(\frac{N_{c}}{T_{N}}\right)P_{\mathrm{FA}} \end{array}$$

where $N_{\mathrm{FA}}$ is the number of false alarms, $T_{N}$ is a predefined signal processing time, and $N_{c}$ is the number of resolution cells collected over $T_{N}$.

In general, the CFAR process operates the detection with a constant false alarm rate or, equivalently, a fixed $P_{\mathrm{FA}}$ given the following key assumptions [1]:

- Background interference is stationary and locally homogeneous allowing statistical moments to be generated temporally and spatially.
- The shape of the PDF for background interference is known.
- A few samples provide a sufficient estimate of the moments to allow the threshold to be set accurately.

**Figure 1:**
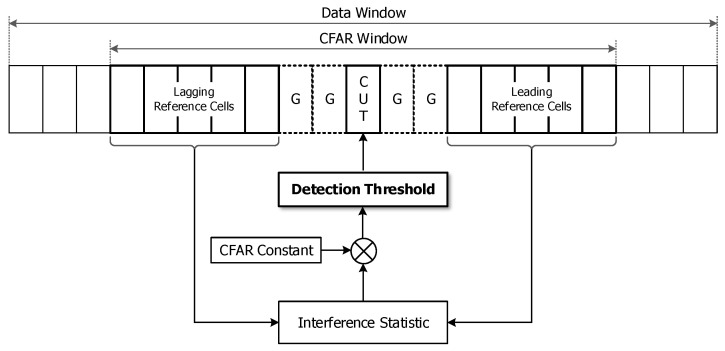
General architecture of the constant false alarm rate (CFAR) process.

The general architecture of the CFAR process is shown in Figure 1. The CFAR window is a subset of the data window and is divided into a cell under test (CUT), guard cells (G), and reference cells. The detection threshold is applied at the CUT that is generally located in the center of the CFAR window, and the signals included in reference cells are used to estimate the statistic of background interference, such as mean, minimum, and median. The CFAR process is executed repeatedly as the CFAR window moves through one cell at an epoch. In general, the detection threshold is determined by the product of the composite interference statistic and the CFAR constant related to $P_{\mathrm{FA},D}$ and a preprocessing type.

The CA-CFAR process determines the detection threshold in estimating the mean value as the interference statistic and selecting the CFAR constant that makes $P_{\mathrm{FA},D}$ for a presumed PDF. If the PDF of background interference is the Rayleigh distribution in voltage like the thermal noise of a system, the PDF of the output at a square-law detector is the exponential PDF and the CA-CFAR constant is found by

(2) $$\begin{array}{c} \alpha_{CA}=N_{s}\;\left[P_{\mathrm{FA},D}^{-1/N_{s}}-1\right] \end{array}$$

where $N_{s}$ is the number of samples within the CFAR window.

In addition, the $P_{\mathrm{FA}}$ of the CA-CFAR process for the Weibull PDF is expressed by

(3) $$\begin{array}{c} P_{\mathrm{FA}}^{CA}={\left[\frac{{\left\{\alpha_{CA}\;f\left(N_{s},c\right)\right\}}^{c}}{N_{s}}+1\right]}^{-N_{s}} \end{array}$$

where $f\left(N_{s},c\right)=\Gamma \left(\frac{1}{c}+1\right)+\left(1-\Gamma \left(\frac{1}{c}+1\right)\right)/N_{s}^{c}$, *c* is the shape parameter of the Weibull PDF, and Γ(·) is the gamma function [14].

If the PDF of background interference is the exponential PDF that coincides to the Weibull PDF with c=1, $P_{\mathrm{FA}}^{CA}$ is equal to $P_{\mathrm{FA},D}$. However, as the PDF becomes the Weibull PDF with a longer tail (c<1), $P_{\mathrm{FA}}^{CA}$ is greater than $P_{\mathrm{FA},D}$ [15]. Moreover, if the detection threshold is manually raised to match $P_{FA}^{CA}$ to a desired level, it will cause a lower $P_{D}$ for a given signal-to-interference ratio since the higher area than the detection threshold for the PDF of the signal and the interference is reduced.

**Figure 2:**
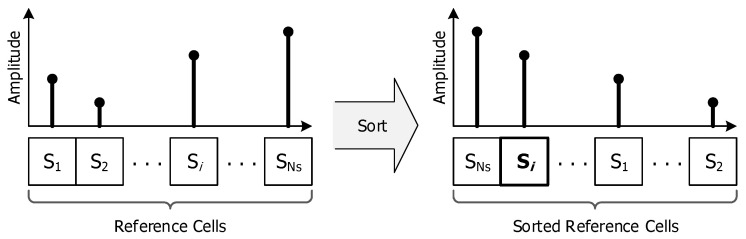
Concept of order statistics constant false alarm rate (OS-CFAR) process.

The OS-CFAR process selects the sample corresponding to a predefined percentile after sorting the reference cells by the amplitude and sets the amplitude of the selected sample to the interference statistic as shown in Figure 2.

Moreover, the OS-CFAR process can adjust $P_{D}$ and $P_{\mathrm{FA}}$ appropriately by changing a percentile for excluding unwanted interference so that Both $P_{D}$ and $P_{\mathrm{FA}}$ will be increased as a lower percentile is chosen and vice versa. The $P_{\mathrm{FA}}$ of the OS-CFAR process for the Weibull PDF is expressed by

(4) $$\begin{array}{c} P_{\mathrm{FA}}^{OS}=\prod_{\ell =0}^{i-1}\frac{N_{s}-\ell }{N_{s}-\ell +{\left(\alpha_{OS}\right)}^{c/2}} \end{array}$$

where $\alpha_{OS}$ is the OS-CFAR constant, and *i* means the *i*-th sample out of $N_{s}$ samples [16].

As the PDF becomes the Weibull PDF with a longer tail (c<1), $P_{\mathrm{FA}}^{OS}$ is also greater than $P_{\mathrm{FA},D}$. In general, the percentile of the OS-CFAR process is selected to be $\left(3N_{s}/4\right)$ for achieving a CFAR loss near the minimum, which causes the OS-CFAR process only to support the suppression of $\left(N_{s}/4\right)$ interference components. Hence, the $P_{\mathrm{FA}}^{OS}$ of the OS-CFAR process can be determined with some degree of robustness compared to that of the CA-CFAR process in the case that the PDF of background interference differs from a presumed PDF.

**Figure 3:**
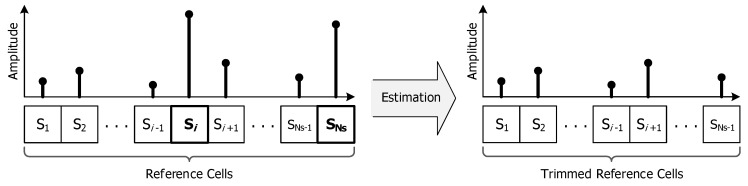
Concept of generalized censored mean level detector (GCMLD) process.

The GCMLD process estimates the information for unwanted interference with the acquired measurements instead of prior knowledge and gets rid of the estimated interference for determining the interference statistic adaptively as shown in Figure 3.

Moreover, both $P_{D}$ and $P_{\mathrm{FA}}$ of the GCMLD process will be increased similarly to the OS-CFAR process as it estimates the information of the unwanted interference more accurately.

A clutter signal is the representative interference that originates from environmental sources, such as terrains, rains, and clouds. Moreover, it has a fundamentally random manner depending on environmental conditions, such as the surface type of the operating region, grazing angle, and local weather. Hence, the PDF of clutter signals is a prior unknown, but is usually known as a kind of exponential PDF with a long tail, such as the Weibull PDF, due to a wide dynamic range of clutter reflection coefficients [3] and thus can be written as

(5) $$\begin{array}{c} p_{C}\left(x\right)=\lambda \;\exp\left(-\lambda \;x\right) \end{array}$$

where λ is the rate parameter of the exponential PDF.

The probability of detecting clutter signals coincides with $P_{\mathrm{FA}}^{c}$ determined by a predefined detection threshold for the PDF of clutter signals as

(6) $$\begin{array}{c} P_{FA}^{c}=\int_{\tau_{k}}^{\infty }p_{C}\left(x\right)dx \end{array}$$

where $\tau_{k}$ is the detection threshold at time *k*.

Therefore, the detection process for clutter signals can be considered as an ill-defined exponential process with strongly nonlinear property and an unknown input-output relationship. Furthermore, $P_{\mathrm{FA}}^{c}$ is relatively small and very sensitive to the detection threshold since the detection process occurs at the tailed area of the PDF as

(7) $$\begin{array}{c} P_{FA{,}_{final}}^{c}={\left(P_{FA{,}_{initial}}^{c}\right)}^{1/\kappa } \end{array}$$

where κ is the final threshold to initial threshold ratio.

In general, the number of the measurements for clutter signals is modeled by the Poisson process as

(8) $$\begin{array}{c} \mu_{F}\left(m_{k}^{c}\right)=\frac{{\left({\bar{m}}_{k}^{c}\right)}^{m_{k}^{c}}\;\exp\left(-{\bar{m}}_{k}^{c}\right)}{m_{k}^{c}!} \end{array}$$

where $m_{k}^{c}$ is the number of the measurements for clutter signals at time *k* and ${\bar{m}}_{k}^{c}$ is the mean number of them at time *k*.

## 3. Robust Control Scheme

The issues of the CFAR process in cluttered environments are as follows:

- The characteristics of clutter signals can be varied temporally and spatially against following a presumed PDF.
- The amplitude of clutter signals has a wide dynamic range.
- The $P_{D}$ for a target signal is decreased as the threshold is increased.

If a feedback structure for controlling the detection threshold is combined with the conventional CFAR process, it can help overcome the fundamental limitation in cluttered environments. The aim of the feedback control for the CFAR process is to adjust the detection threshold to minimize the error of $P_{\mathrm{FA}}$ caused by the model difference between a presumed PDF and a true PDF of clutter signals. Moreover, the controller should have the capability of robustness, rather than precision, due to the dynamic range of clutter signals.

**Figure 4:**
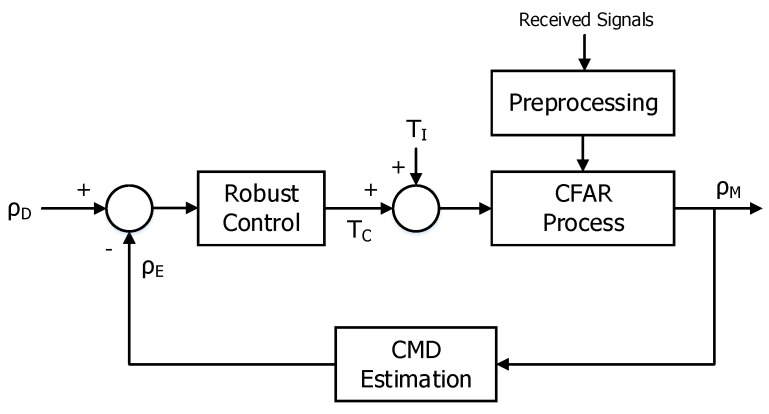
Structure of the proposed robust control scheme.

We propose the robust control scheme for tuning the detection threshold of the CFAR process in cluttered environments on the assumption that a presumed PDF of clutter signals is an exponential type but the shape and parameters for a true PDF are unknown, which consists of the preprocessing, the CFAR process, the CMD estimation, and the robust control as depicted in Figure 4.

The preprocessing executes the integration for received signals and then the CFAR process performs the detection for the output of the preprocessing with an ideal detection threshold ($T_{I}$) at the initial epoch. The CMD estimation calculates an estimated CMD ($\rho_{E}$) from a measured CMD ($\rho_{M}$) based on the output of the CFAR process. The robust control generates an additive detection threshold ($T_{C}$) based on the difference between a desired CMD ($\rho_{D}$) and an estimated CMD which is related to the error of $P_{\mathrm{FA}}$ for the PDF. Therefore, the detection threshold of the CFAR process will be adjusted automatically until $\rho_{E}$ equals to $\rho_{D}$. In addition, any CFAR process, including the latest CFAR process as well as the conventional CFAR process with an open loop structure in terms of $P_{FA}$, can be applied to the proposed structure and a designer can set $\rho_{D}$ related to $P_{\mathrm{FA},D}$ to an affordable level of a radar system in cluttered environments. It can also be used in clutter-free environments by considering an ideal $P_{\mathrm{FA},D}$ based on the PDF of a system noise, and then $T_{C}$ may be zero. Therefore, a radar system will become more robust if a designer manages the level of $\rho_{D}$ adaptively as the condition of an operating environment. For example, in case of operating the medium pulse repetition frequency (PRF) waveform mode, a designer can normally set $\rho_{D}$ to a lower level because the system tries to locate a target signal into a clutter-free region by selecting an optimized PRF. However, the situation that clutter signals are included in an ROI can happen owing to the encounter angle and maneuvering property of a target and the hardware limitation for the range of PRF. Moreover, the PDF of clutter signals in the ROI may be very different from a presumed PDF because of the folding effect of a PRF for clutter signals. By adjusting $\rho_{D}$ to a higher level, the radar system can maintain the detection performance adaptively in the situation.

### 3.1. Preprocessing for Received Signals

The amplitude of clutter signals can fluctuate by more than two orders of amplitude. Owing to the original nature of clutter signals, it can be difficult to control the detection threshold stably and the $P_{D}$ for the target signal can severely be decreased though $P_{\mathrm{FA},D}$ is achieved. The preprocessing, such as coherent and noncoherent integration, improves the detection performance. The coherent integration can basically be applied for increasing $P_{D}$. Moreover, the noncoherent integration affects the variance for the PDF of the clutter signals so that it can reduce $P_{\mathrm{FA}}$ and raise $P_{D}$ indirectly. It is well known that the limited sum for the random samples of the exponential PDF becomes a random variable with the Erlang PDF given that the samples are independent identically distributed as

(9) $$\begin{array}{c} p_{e}\left(z\right)=\sum_{\ell =1}^{N_{p}}\lambda \exp\left(-\lambda \;x_{\ell }\right)=\frac{\lambda^{N_{p}}\;z^{N_{p}-1}\;\exp\left(-\lambda z\right)}{(N_{p}-1)!} \end{array}$$

where $N_{p}$ is the number of samples for the noncoherent integration.

**Figure 5:**
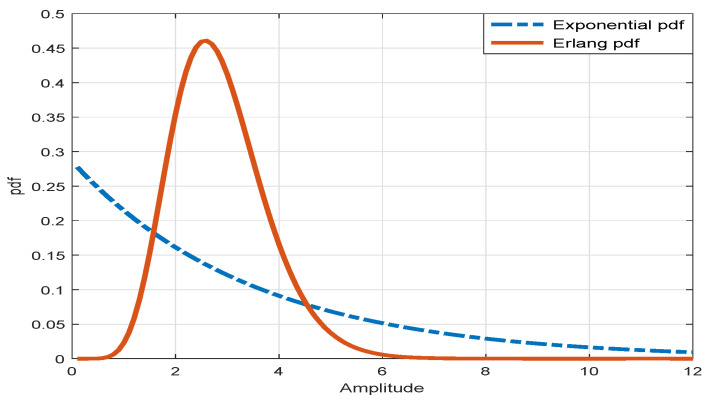
Relationship of exponential and Erlang probability density function (PDF) ($N_{p}=10$).

Figure 5 shows that the long tail of the exponential PDF becomes shortened via the sum for the samples of the exponential PDF.

In addition, the exponential PDF is transformed to the Gaussian PDF as the number of samples becomes larger ($N_{p}\to \infty$) based on the central limit theorem, and thus the variance of the PDF is dramatically reduced. However, in actual applications, there will be limitations for increasing the number of samples owing to factors, such as range walk of target and signal processing load. Consequently, the preprocessing chain with the sequential combination of the coherent and the noncoherent integration, which is a design parameter in a radar system, can be helpful for decreasing the dynamic range of clutter signals and improving the detection performance for a target signal.

### 3.2. CMD Estimation

The CMD is proportional to the $P_{\mathrm{FA}}$ from clutter signals and can be estimated at time *k* by

(10) $$\begin{array}{c} {\hat{\rho }}_{k}=\frac{{\hat{m}}_{k}^{c}}{V_{k}}=C_{\mathrm{FA}}^{\rho }\;P_{\mathrm{FA}} \end{array}$$

where ${\hat{m}}_{k}^{c}$ is the estimated number of the measurements for clutter signals within an ROI, $V_{k}$ is the volume of the ROI that is typically defined by the covariance information of predicted states, and $C_{\mathrm{FA}}^{\rho }$ is the constant determined by the design parameters of a system.

The standard estimator for the CMD based on the PTE is proposed in the integrated probabilistic data association (IPDA) filter [8] as

(11) $$\begin{array}{c} {\hat{\rho }}_{k}=\frac{1}{V_{k}}\left[m_{k}-P_{D}P_{G}\;P\left(\chi_{k}|Z^{k-1}\right)\right] \end{array}$$

where $m_{k}$ is the number of the measurements for received signals at time *k*, $P_{G}$ is the probability that there exists a target signal within an ROI, $\chi_{k}$ is the event that there exists a target within an ROI at time *k*, $Z^{k-1}$ is the sequence of all measurement sets from the initial time to time k−1; $Z^{k-1}=\{z_{k-1}⋃Z^{k-2}$}, and $P(\chi_{k}|Z^{k-1})$ means the predicted PTE with all measurement sets up to time k−1.

It may be speculated that the estimated number of the measurements for clutter signals equals the difference between the number of the measurements for received signals and the estimated number of the measurement for a target signal that is expressed by the probability for the joint event which a target exists, a target signal falls into an ROI, and a target signal is detected given that the number of a target signal is only one and the rest of the signals originate from clutter. It may yield a biased output, particularly in the case that there is no target within an ROI [17].

The conditional mean estimator for the CMD based on the probability of target perceivability, in which a target is perceivable if it exists and is detectable, is proposed [12] and is expressed by

(12) $$\begin{array}{c} {\hat{\rho }}_{k}=E\left[\rho_{k}|Z^{k}\right]=\frac{1}{V_{k}}E\left(m_{k}^{c}|Z^{k}\right)=\frac{1}{V_{k}}\left[m_{k}-E\left(m_{k}^{T}|Z^{k}\right)\right]=\frac{1}{V_{k}}\left[m_{k}-\frac{r_{k}\sigma_{k}}{{\hat{\rho }}_{k}+r_{k}\sigma_{k}}\right] \end{array}$$

with

(13) $$r_{k}=\frac{P_{D}P_{G}P\left(O_{k}|Z^{k-1}\right)}{1-P_{D}P_{G}P\left(O_{k}|Z^{k-1}\right)}\;,\;\sigma_{k}=p\left(z_{k}|m_{k}^{T}=1,Z^{k-1}\right)$$

where $m_{k}^{T}$ is the number of the measurement for a target signal, $\sigma_{k}$ is the likelihood function for $z_{k}$ given that the number of the measurement for a target signal is equal to one and the acquired measurement sets are $\left\{Z^{k-1}\right\}$, and $P(O_{k}|Z^{k-1})$ denotes the predicted probability of target perceivability with all measurement sets up to time k−1.

The probability of target perceivability is conceptually analogous to the PTE with a Markov chain one model for the propagation of target existence. Although the notion of target perceivability rigorously differs from that of target existence, target existence implies target detectability in a Markov chain one model. In a Markov chain two model, on the other hand, target existence and target detectability are so separated that the condition that a target exists and is nondetectable can be considered [18]. The conditional mean estimator is distribution free and theoretically yields an unbiased result using all measurements up to time *k*. However, there exists the contradiction that for calculating ${\hat{\rho }}_{k}$, ${\hat{\rho }}_{k}$ must be known in advance. Hence, an additional iteration process has to be required to obtain a practical solution.

The maximum likelihood estimator for the CMD is also proposed given that the CMD at each time is unknown but nonrandom [12].

(14) $$\begin{array}{c} {\hat{\rho }}_{k}=\arg\;\max_{\rho_{k}>0}\;p\left(z_{k}|\rho_{k},Z^{k-1}\right)=\frac{m_{k}}{2\;V_{k}}[\left(1-r_{k}^{\sigma }\right)+\sqrt{{\left(1-r_{k}^{\sigma }\right)}^{2}+4\;\frac{m_{k}-1}{m_{k}}\;r_{k}^{\sigma }}] \end{array}$$

with

(15) $$r_{k}^{\sigma }=r_{k}\sigma_{k}V_{k}/m_{k}$$

It can be the complementary estimator to provide the initial estimate required by another estimator as well as the primary estimator for the CMD. It can yield a biased result in practice since it is required to set a lower limit value in order to prevent a numerical error and very difficult to be well integrated with a tracking filter.

**Figure 6:**
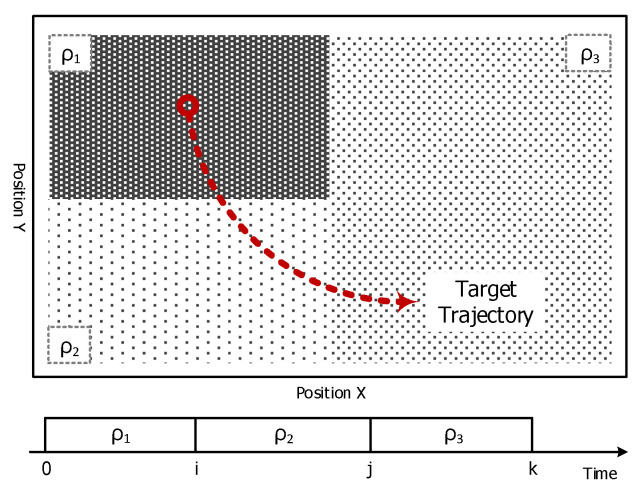
Property of the clutter measurement density (CMD) in a heterogeneous region.

These track-oriented CMDEs fundamentally use all past observations from the initial time to the current time. However, the CMD may be changed in time, especially in a heterogeneous region as shown in Figure 6, such that CMDs related to each measurement sets can be different for each other and the only measurement sets over a limited time duration will be the meaningful information for estimating the current CMD.

To the best of the authors’ knowledge, it is reasonable to estimate the current CMD using the measurement sets over the finite time interval on the assumption that CMD is a constant during a short time. Therefore, we propose the CMDE based on conditional mean with finite measurement sets as follows:

(16) $$\begin{array}{c} {\hat{\rho }}_{k}^{N}≜E\left[\rho_{k}|Z^{k-N:k-1}\right]=\frac{1}{V_{k}}\left[{\bar{m}}_{k}-P_{D}P_{G}\;P\left(\chi_{k}|Z^{k-N:k-1}\right)\right] \end{array}$$

with

(17) $${\bar{m}}_{k}=E\left[m_{k}|Z^{k-N:k-1}\right]=\frac{1}{N}\sum_{\ell =1}^{N}m_{k-\ell }$$

where *N* is the size of finite measurement sets, ${\bar{m}}_{k}$ is the sample mean for the number of measurement sets at time *k*, and $P(\chi_{k}|Z^{k-N:k-1})$ denotes the predicted PTE with finite measurement sets.

The PTE with finite measurement sets must be derived for calculating the proposed CMDE. First, the predicted PTE with finite measurement sets is simply obtained by applying the propagation matrix of the Markov chain one model as

(18) $$\begin{array}{cc} \; & \left[\begin{array}{c} P(\chi_{k}|Z^{k-N:k-1})\\ P({\bar{\chi }}_{k}|Z^{k-N:k-1}) \end{array}\right]={\left[\begin{array}{c} \pi_{11}\;\pi_{12}\\ \pi_{21}\;\pi_{22} \end{array}\right]}^{T}\;\left[\begin{array}{c} P(\chi_{k-1}|Z^{k-N:k-1})\\ P({\bar{\chi }}_{k-1}|Z^{k-N:k-1}) \end{array}\right] \end{array}$$

where ${\bar{\chi }}_{k}$ is the event that there is no target within an ROI at time *k* and $\pi_{ij}\;\left(i,j\in \left\{1,2\right\}\right)$ is the element of the matrix for the transitional probabilities between the states of target existence with the constraint: $\pi_{11}+\pi_{12}=\pi_{21}+\pi_{22}=1$.

It is generally recommended to set $\pi_{21}=0$ and $\pi_{22}=1$ since the value of $\pi_{21}$ means the transitional probability that a false target turns into a true target, so the issue is not in the tracking phase but in the initialization phase of a tracking filter [9].

On the assumption that the measurement sets $\left\{Z^{k-N}\right\}$ and $\left\{Z^{k+1-N:k}\right\}$ are conditionally independent given $\chi_{k}$ or ${\bar{\chi }}_{k}$ because all past events of target existence can be known by the current event of target existence via the backward Markov chain propagation matrix [19], the PTE with the measurement sets up to time *k* can be expressed as follows:

(19) $$\begin{array}{cc} P(\chi_{k}|Z^{k}) & =P\left(\chi_{k}|Z^{k-N},Z^{k+1-N:k}\right)=\frac{p\left(Z^{k-N}|\chi_{k}\right)\;p\left(Z^{k+1-N:k}|\chi_{k}\right)\;P\left(\chi_{k}\right)}{p(Z^{k})}\\ \; & =\left\{\frac{P\left(\chi_{k}|Z^{k-N}\right)\;P\left(\chi_{k}|Z^{k+1-N:k}\right)}{P(\chi_{k})}\right\}\left\{\frac{p\left(Z^{k-N}\right)\;p\left(Z^{k+1-N:k}\right)}{p(Z^{k})}\right\} \end{array}$$

Hence, the PTE with the measurement sets from time k+1−N to time *k* is simply expressed by

(20) $$\begin{array}{c} P\left(\chi_{k}|Z^{k+1-N:k}\right)=\left\{\frac{P\left(\chi_{k}|Z^{k}\right)\;P\left(\chi_{k}\right)}{P(\chi_{k}|Z^{k-N})}\right\}\left\{\frac{p(Z^{k})}{p\left(Z^{k-N}\right)\;p\left(Z^{k+1-N:k}\right)}\right\} \end{array}$$

Moreover, the ratio of the probability of target existence to the probability of target non-existence with the measurement sets from time k+1−N to time *k* is written as

(21) $$\begin{array}{c} \frac{P(\chi_{k}|Z^{k+1-N:k})}{P({\bar{\chi }}_{k}|Z^{k+1-N:k})}=\frac{P(\chi_{k}|Z^{k})}{P({\bar{\chi }}_{k}|Z^{k})}\frac{P({\bar{\chi }}_{k}|Z^{k-N})}{P(\chi_{k}|Z^{k-N})}\left[\frac{P(\chi_{k})}{P({\bar{\chi }}_{k})}\right] \end{array}$$

To build the recursive formula, the ratio of the probability of target existence to the probability of target non-existence with the measurement sets from time k−N to time k−1 is expressed by

(22) $$\begin{array}{cc} \; & \frac{P(\chi_{k}|Z^{k-N:k-1})}{P({\bar{\chi }}_{k}|Z^{k-N:k-1})}=\frac{P(\chi_{k}|Z^{k-1})}{P({\bar{\chi }}_{k}|Z^{k-1})}\frac{P({\bar{\chi }}_{k}|Z^{k-1-N})}{P(\chi_{k}|Z^{k-1-N})}\left[\frac{P(\chi_{k})}{P({\bar{\chi }}_{k})}\right] \end{array}$$

Finally, the relationship between each ratio of the probability of target existence to the probability of target non-existence is derived via combining Equation (21) with Equation (22), and it can also be written as the prevalent Formula (24).

(23) $$\begin{array}{cc} \frac{P(\chi_{k}|Z^{k+1-N:k})}{P({\bar{\chi }}_{k}|Z^{k+1-N:k})} & =\left[\frac{P(\chi_{k}|Z^{k})}{P({\bar{\chi }}_{k}|Z^{k})}\frac{P({\bar{\chi }}_{k}|Z^{k-1})}{P(\chi_{k}|Z^{k-1})}\right]{\left[\frac{P(\chi_{k}|Z^{k-N})}{P({\bar{\chi }}_{k}|Z^{k-N})}\frac{P({\bar{\chi }}_{k}|Z^{k-1-N})}{P(\chi_{k}|Z^{k-1-N})}\right]}^{-1}\frac{P(\chi_{k}|Z^{k-N:k-1})}{P({\bar{\chi }}_{k}|Z^{k-N:k-1})}\\ & =\left(\frac{\Lambda_{k}}{\Psi_{k-N}}\right)\frac{P(\chi_{k}|Z^{k-N:k-1})}{P({\bar{\chi }}_{k}|Z^{k-N:k-1})} \end{array}$$

Here, $\Lambda_{k}$ is the measurement likelihood ratio at time *k* [20] and $\Psi_{k-N}$ is the function of the Markov chain propagation matrix, the measurement likelihood ratio, and the PTE at time k−N.

(24) $$\begin{array}{c} P\left(\chi_{k}|Z^{k+1-N:k}\right)=\frac{\left(\frac{\Lambda_{k}}{\Psi_{k-N}}\right)P\left(\chi_{k}|Z^{k-N:k-1}\right)}{P\left({\bar{\chi }}_{k}|Z^{k-N:k-1}\right)+\left(\frac{\Lambda_{k}}{\Psi_{k-N}}\right)P\left(\chi_{k}|Z^{k-N:k-1}\right)} \end{array}$$

The measurement likelihood ratio at time *k* is expressed by

(25) $$\begin{array}{c} \Lambda_{k}=\left(1-P_{D}P_{G}\right)+P_{D}P_{G}\sum_{i=1}^{m_{k}}\frac{N(z_{k,i}\;;{\bar{z}}_{k},S_{k})}{\rho_{k}P_{G}} \end{array}$$

where N(·) is the Gaussian PDF, $z_{k,i}$ is *i*-th measurement at time *k*, and ${\bar{z}}_{k}$ and $S_{k}$ are the predicted mean and covariance for measurement of the tracking filter.

The function $\Psi_{k-N}$ is expressed as follows; the detailed derivation is shown in the Appendix A.

(26) $$\begin{array}{cc} \Psi_{k-N} & =\left(\Lambda_{k-N}\right)\frac{\left[\frac{1}{1-{\left(\pi_{11}\right)}^{N}}+\frac{P(\chi_{k-N}|Z^{k-1-N})}{P({\bar{\chi }}_{k-N}|Z^{k-1-N})}\right]}{\left[\frac{1}{1-{\left(\pi_{11}\right)}^{N}}+\frac{P(\chi_{k-N}|Z^{k-N})}{P({\bar{\chi }}_{k-N}|Z^{k-N})}\right]} \end{array}$$

Here, $\Lambda_{k-N}$ is the measurement likelihood ratio at time k−N.

Note that it has a similar form to the PTE with the measurement sets from the initial time to time *k* except for the term of the measurement likelihood ratio. It can be seen that for updating the PTE with finite measurement sets at time *k*, the information for the obtained measurement at time *k* is exploited, but the information for the acquired measurement and the PTE at time k−N is discarded after being propagated up to time *k*.

(27) $$\begin{array}{c} P\left(\chi_{k}|Z^{k}\right)=\sum_{i=0}^{m_{k}}P\left(\theta_{k,i},\chi_{k}\;|\;z_{k},m_{k},Z^{k-1}\right)=\frac{\Lambda_{k}\;P\left(\chi_{k}|Z^{k-1}\right)}{P\left({\bar{\chi }}_{k}|Z^{k-1}\right)+\Lambda_{k}\;P\left(\chi_{k}|Z^{k-1}\right)} \end{array}$$

Here, $\theta_{k,i}$ is the associated event that the *i*-th measurement of $z_{k}$ originates from the target and the rest are from clutter at time *k*.

### 3.3. Robust Control

Despite the preprocessing for received signals, the amplitude of the signals can still fluctuate somewhat owing to the limit for the number of the integration and the correlation degree of samples [21]. The scintillation effect will be considered as a disturbance input for controlling the detection threshold toward $P_{\mathrm{FA},D}$ from the viewpoint of the control concept. Therefore, it is reasonable to apply the robust controller to minimize the effect of the disturbance for the CFAR process.

In general, proportional-integral-derivative (PID) control is the most intuitive control method due to clearly physical meanings. It can be used irrespective of system dynamics so that it has been widely accepted in industry. For applying the PID controller to the CFAR process, the state vector of the difference for CMD is defined by

(28) $$\begin{array}{c} x≜{\left[\int e\;dt\;e\;\dot{e}\right]}^{T}\in {ℜ}^{3},\;e≜\rho_{D}-\rho_{E} \end{array}$$

As mentioned before, the system dynamics of the state vector will have a nonlinear function with unknown parameters. On the assumption that the system modeling errors caused by the difference between a true PDF and an exponential PDF presumed for clutter signals and linearizing the nonlinear system are considered as a disturbance term, the linear system dynamics of the state vector can be expressed by

(29) $$\begin{array}{c} \dot{x}=A\left(t\right)x+Bu+Bw \end{array}$$

with

(30) $$A\left(t\right)=\left[\begin{array}{ccc} 0 & 1 & 0\\ 0 & 0 & 1\\ a_{31} & a_{32} & a_{33} \end{array}\right],\;B=\left[\begin{array}{c} 0\\ 0\\ 1 \end{array}\right]$$

where *u* is a control input, *w* is a disturbance input, and $\left[a_{31},a_{32},a_{33}\right]$ are arbitrary variables.

To minimize the effect of the disturbance for the given system with Equation (29), the $H_{\infty }$ performance index has to be defined by

(31) $$\begin{array}{c} J\left(x,u,w,t\right)=\lim_{t\to \infty }\left[V\left(x,t\right)+\frac{1}{2}\int_{0}^{t}\left(x^{T}Qx+u^{T}Ru-\gamma^{2}w^{T}w\right)d\sigma \right] \end{array}$$

where V(x,t) is the Lyapunov function, *Q* is a state weighting matrix, *R* is a control input weighting matrix, and γ is the $L_{2}$ gain related to the disturbance suppression.

The Hamilton–Jacobi–Isaacs (HJI) equation is generally derived from the optimization for the $H_{\infty }$ performance index. Both the optimal control input, $H_{\infty }$ control, and the allowable maximal disturbance input can be determined after finding the Lyapunov function satisfied with the HJI equation, but it is very difficult to obtain the solution.

(32) $$\begin{array}{c} u^{*}=-R^{-1}B^{T}\;V_{x}^{T},\;w^{*}=\frac{1}{\gamma^{2}}\;B^{T}\;V_{x}^{T} \end{array}$$

Hence, the theorem of inverse optimal PID control can help overcome such difficulty. First, the control input weighting matrix and the Lyapunov function are defined with the constraints: $K>0,\gamma >0,P>0,K_{I}>0,K_{P}>0$.

(33) $$\begin{array}{c} R≜{\left(K+\frac{1}{\gamma^{2}}\right)}^{-1},\;V(x,t)≜\frac{1}{2}x^{T}Px \end{array}$$

with

(34) $$P=\left[\begin{array}{ccc} K_{I}^{2}+K_{I}K_{P}K & K_{I}K_{P}+K_{I}K & K_{I}\\ K_{P}K_{I}+K_{I}K & K_{P}^{2}+K_{P}K & K_{P}\\ K_{I} & K_{P} & 1 \end{array}\right]$$

As a result, the HJI equation is converted into the algebraic Riccati equation for any x≠0, and the state weighting matrix Q(>0) can be found as a solution.

(35) $$\begin{array}{c} A^{T}P+PA-PBR^{-1}B^{T}P+\frac{1}{\gamma^{2}}PBB^{T}P+Q=0 \end{array}$$

If the matrix Q exists with some conditions, then the control input can be expressed as the form of a PID controller.

(36) $$\begin{array}{c} u=-\left(K+\frac{1}{\gamma^{2}}I\right)\left[\dot{e}+K_{P}e+K_{I}\int e\;dt\right] \end{array}$$

Furthermore, the closed loop control system is extended disturbance input-to-state stable based on the following condition [22,23].

(37) $$\begin{array}{c} \dot{V}\leq -\frac{1}{2}x^{T}\left(Q+PBKB^{T}P\right)x+\gamma^{2}{\left|w\right|}^{2} \end{array}$$

Note that the matrix Q is the function of the elements of the matrix A and the gains of the controller so that it will satisfy the Riccati equation with a limited range for each element of matrix A given that the gains are fixed. For the example of K=5, $K_{P}=15,K_{I}=0.15,\gamma =1$, the element $a_{31}$ is in the range from −16.5 to −0.1, the element $a_{32}$ is in the range from −1030 to −10, and the element $a_{33}$ is in the range from −124 to −16 for the solution. In addition, the larger the value of γ, the smaller the allowable maximal disturbance input, i.e., the robustness of a control system for a disturbance is decreased.

## 4. Simulation Results

The simulation experiments are divided into the section for verifying the performance of the proposed PTE and CMDE, and the section for evaluating the whole performance of the robust control scheme in cluttered environments. In addition, the standard estimator for the CMD is applied as the conventional CMDE to compare the features of the PTE and the CMDE together; the OS-CFAR process is applied for the performance comparison because it is representative method for a single target in cluttered environments; the IPDA filter for relative velocity and acceleration of a target is used; the time step for the simulation is 2 msec; the design parameters for PTE and CMDE are set to $P_{D}=0.9$, $P_{G}=0.99$, $\pi_{11}=0.98$, and N=25.

First, the main conditions of the scenario for confirming the performance of the proposed PTE and CMDE are as follows:

- A single target with the constant velocity moves in the cluttered region and the target signal is detected over (0.2–0.95) s.
- The number of detection for clutter signals is determined by the Poisson process and the CMD is changed discretely in time: (0.06→0.005→0.04)

**Figure 7:**
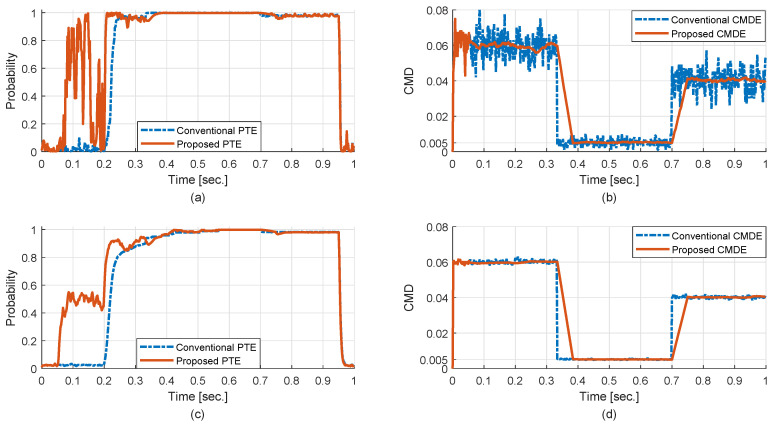
Performance of the probability of target existence (PTE) and the CMD estimation (CMDE): (**a**) PTE via 1 Monte Carlo (MC) run, (**b**) CMDE via 1 MC run, (**c**) PTE via 100 MC runs, (**d**) CMDE via 100 MC runs.

Figure 7 presents the features of the PTE and the CMDE. From the simulation results via one Monte Carlo (MC) run, as shown in Figure 7a,b, it can be seen that the proposed PTE has a faster response than the conventional one at the instant that the status of target existence is changed, and the proposed CMDE has less error variance than the conventional one owing to the effect of sample mean, though a little latency exists in the transition interval of the CMD. Furthermore, from the simulation results via 100 MC runs, as shown in Figure 7c,d, it can also be confirmed that the proposed method has a more sensitive response and an accuracy of estimation with less error variance than the conventional one. Note that as *N* is increased, the characteristics of the proposed PTE become similar to those of the conventional PTE, and the error variance is decreased and the latency is increased for the proposed CMDE. Moreover, when the proposed PTE has a higher value randomly in the situation that only clutter signals exist within the ROI, it can be ignored in the confirmation process of track management.

**Figure 8:**
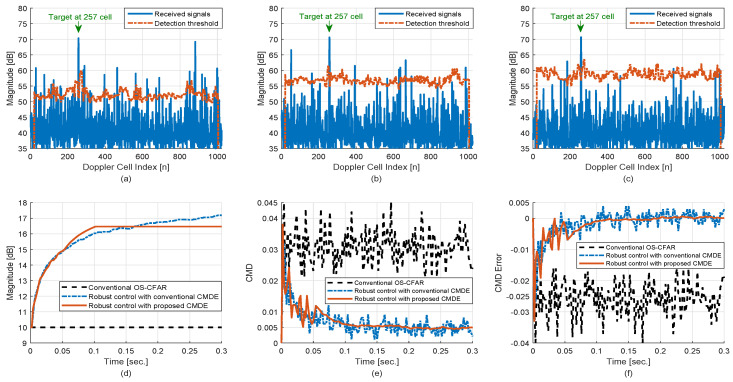
Performance of the robust control scheme in the constant CMD (1 MC run): (**a**) detection of the proposed method at 0.0 s, (**b**) detection of the proposed method at 0.05 s, (**c**) detection of the proposed method at 0.1 s, (**d**) CFAR constant, (**e**) CMDE, (**f**) CMD error.

**Figure 9:**
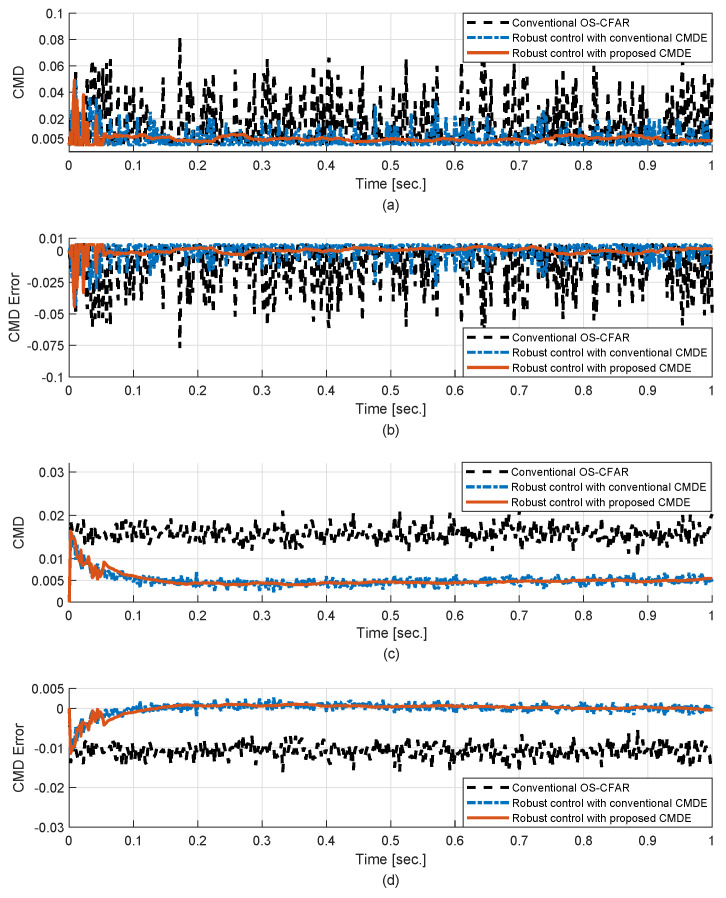
Performance of the robust control scheme in the random CMD: (**a**) CMDE via 1 MC run, (**b**) CMD error via 1 MC run, (**c**) CMDE via 100 MC runs, (**d**) CMD error via 100 MC runs.

The principal conditions of the scenario for ascertaining the performance of the proposed robust control scheme are as follows:

- The number of the noncoherent integration is set to $N_{p}=5$.
- The OS-CFAR process is applied with the percentile of 0.75 and $P_{\mathrm{FA},D}=5\times 10^{-3}($$=\rho_{D})$.
- A single target with the constant velocity moves in the cluttered region and the target signal is generated with the Swerling 1 model: (Doppler cell index = 257)
- The amplitude of clutter signals has the property of the Weibull PDF, the number of detection is dependent on the CFAR process, and the position of detection is uniformly distributed in the Doppler frequency domain as the grazing angle of clutter.
- The CMD is the constant (0.03, Figure 8) or varied randomly in time (Figure 9).
- The design parameters for the robust PID controller are set to K=5, $K_{P}=15,K_{I}=0.15,\gamma =1$ with the constraint: The CFAR constant is maintained at the last tuned level if the error of the CMD stays within $\pm (P_{\mathrm{FA},D}/5)$.

The performance of the robust control scheme in the constant CMD situation is depicted in Figure 8. For each epoch, the CFAR process is executed for the preprocessed signals in the Doppler frequency domain as shown in Figure 8a–c and the number of detection is determined by the CFAR process. Then the CMD is estimated using the number of detection and the PTE as shown in Figure 8e, and the CFAR constant, one of the parameters for determining detection threshold, is adjusted as shown in Figure 8d to reduce the error between $\rho_{D}$ and $\rho_{E}$, as shown in Figure 8f. In the case that the OS-CFAR process is only applied without the robust control scheme, the CMD error could not be minimized since the CFAR constant remained at the initial value in time. In addition, in the case that the robust control scheme is applied with the conventional CMDE, the CMD error was minimized with relatively large variance so that the control loop could not be stabilized easily and the CFAR constant was increased continually. However, in the case that the robust control scheme is applied with the proposed CMDE, the CMD error was minimized and stabilized within about 0.1 s. due to the constraint of the controller as the CFAR constant was adjusted from 10 dB to 16.5 dB automatically. Furthermore, the robust control scheme with the proposed CMDE improved the tracking performance of the IPDA filter considerably because the effect of clutter signals was dramatically suppressed as the CMD was decreased rapidly and stably to the desired level.

Figure 9 shows the performance of the robust control scheme in the time-varying CMD situation with the severe condition that the parameter of the Weibull PDF is changed for each time with the uniform PDF over (0.3–0.6) [24]. It is presumed that in a heterogeneous cluttered region, the amplitude statistic of ground clutter can be expressed by the Weibull PDF and the parameter linked with $P_{\mathrm{FA}}$ can be varied continuously within a specific range. In the case that the OS-CFAR process is only applied without the robust control scheme, the CMD error could not be minimized. However, in the case that the robust control scheme is applied, the CMD error was minimized and the control system tried to be stabilized as the CFAR constant was tuned continually though the CMD was changed randomly in time. Furthermore, it can be seen that the robust control scheme with the proposed CMDE has less error variance than that of the conventional one.

## 5. Conclusions

It is a crucial point to maintain the probability of false alarm at a desired level in radar systems. Although several CFAR processes have been researched for achieving this purpose in cluttered environments, it may be tricky for them to be applied since the amplitude of clutter signals has severely dynamic characteristics and they fundamentally have an open loop structure in terms of $P_{FA}$. This paper presents the robust control scheme for adjusting the detection threshold of the CFAR process, in which for applying the feedback structure, the CMDE method based on the newly derived PTE with finite measurement sets is proposed. Furthermore, it is shown that the proposed method has superior robustness and adaptiveness in various cluttered environments through the simulation experiments, in which the CMD was controlled robustly to the desired level by automatically adjusting the CFAR constant of the CFAR process for the constant and random CMD situations. It is anticipated that it will be expandable to the applications of the multiple target tracking filters and multiple model tracking filters based on the PTE and it can be a feasible solution in actual applications, especially in a heterogeneous cluttered environment. Further studies will be required to optimize the size of finite measurement sets for the PTE and the CMDE. 

## Abbreviations

The following abbreviations are used in this manuscript:

CA-CFAR	Cell-averaging constant false alarm rate
CFAR	Constant false alarm rate
CMD	Clutter measurement density
CMDE	Clutter measurement density estimation
CUT	Cell under test
GCMLD	Generalized censored mean level detector
HCE-CFAR	Heterogeneous clutter estimating constant false alarm rate
HJI	Hamilton–Jacobi–Isaacs
IPDA	Integrated probabilistic data association
MC	Monte Carlo
OS-CFAR	Order statistics constant false alarm rate
PDF	Probability density function
PID	Proportional-integral-derivative
PRF	Pulse repetition frequency
PTE	Probability of target existence
ROI	Region of interest

## Appendix A

To complete the recursive form of the PTE with finite measurement sets, the function $\Psi_{k-N}$ must be derived.

(A1) $$\begin{array}{c} \Psi_{k-N}≜\frac{P(\chi_{k}|Z^{k-N})}{P({\bar{\chi }}_{k}|Z^{k-N})}\frac{P({\bar{\chi }}_{k}|Z^{k-1-N})}{P(\chi_{k}|Z^{k-1-N})} \end{array}$$

The probability $P(\chi_{k}|Z^{k-N})$ means that the predicted probability of target existence at time *k* with measurement sets $\left\{Z^{k-N}\right\}$, and it can be expressed as follows:

(A2) $$\begin{array}{cc} \left[\begin{array}{c} P(\chi_{k}|Z^{k-N})\\ P({\bar{\chi }}_{k}|Z^{k-N}) \end{array}\right] & =\left[\begin{array}{cc} \pi_{11} & 0\\ 1-\pi_{11} & 1 \end{array}\right]\;\;\;\;\;\left[\begin{array}{c} P(\chi_{k-1}|Z^{k-N})\\ P({\bar{\chi }}_{k-1}|Z^{k-N}) \end{array}\right]\\ \; & ={\left[\begin{array}{cc} \pi_{11} & 0\\ 1-\pi_{11} & 1 \end{array}\right]}^{2}\;\;\left[\begin{array}{c} P(\chi_{k-2}|Z^{k-N})\\ P({\bar{\chi }}_{k-2}|Z^{k-N}) \end{array}\right]\\ \; & \;\;\;\;\;\;\;\;\;\vdots \\ \; & ={\left[\begin{array}{cc} \pi_{11} & 0\\ 1-\pi_{11} & 1 \end{array}\right]}^{N}\left[\begin{array}{c} P(\chi_{k-N}|Z^{k-N})\\ P({\bar{\chi }}_{k-N}|Z^{k-N}) \end{array}\right] \end{array}$$

Similarly, $P(\chi_{k}|Z^{k-1-N})$ is also expressed by

(A3) $$\begin{array}{c} \left[\begin{array}{c} P(\chi_{k}|Z^{k-1-N})\\ P({\bar{\chi }}_{k}|Z^{k-1-N}) \end{array}\right]=\left[\begin{array}{c} {\left(\pi_{11}\right)}^{N}\;\;\;\;\;\;0\\ 1-{\left(\pi_{11}\right)}^{N}\;1 \end{array}\right]\;\left[\begin{array}{c} P(\chi_{k-N}|Z^{k-1-N})\\ P({\bar{\chi }}_{k-N}|Z^{k-1-N}) \end{array}\right] \end{array}$$

In addition, the ratio of the probability of target existence to the probability of target non-existence at time k−N is written as

(A4) $$\begin{array}{c} \frac{P(\chi_{k-N}|Z^{k-N})}{P({\bar{\chi }}_{k-N}|Z^{k-N})}=\left(\Lambda_{k-N}\right)\frac{P(\chi_{k-N}|Z^{k-1-N})}{P({\bar{\chi }}_{k-N}|Z^{k-1-N})} \end{array}$$

By substituting Equations (A2)–(A4) into Equation (A1), the function $\Psi_{k-N}$ is finally derived as follows:

(A5) $$\begin{array}{cc} \Psi_{k-N}= & \;\left[\frac{{\left(\pi_{11}\right)}^{N}\;P\left(\chi_{k-N}|Z^{k-N}\right)}{\left\{1-{\left(\pi_{11}\right)}^{N}\right\}\;P\left(\chi_{k-N}|Z^{k-N}\right)+P\left({\bar{\chi }}_{k-N}|Z^{k-N}\right)}\right]\times \\ \; & \;\left[\frac{\left\{1-{\left(\pi_{11}\right)}^{N}\right\}\;P\left(\chi_{k-N}|Z^{k-1-N}\right)+P\left({\bar{\chi }}_{k-N}|Z^{k-1-N}\right)}{{\left(\pi_{11}\right)}^{N}\;P\left(\chi_{k-N}|Z^{k-1-N}\right)}\right]\\ = & \;\frac{1-{\left(\pi_{11}\right)}^{N}+\frac{P({\bar{\chi }}_{k-N}|Z^{k-1-N})}{P(\chi_{k-N}|Z^{k-1-N})}}{1-{\left(\pi_{11}\right)}^{N}+\frac{P({\bar{\chi }}_{k-N}|Z^{k-N})}{P(\chi_{k-N}|Z^{k-N})}}=\frac{\left\{1-{\left(\pi_{11}\right)}^{N}\right\}\frac{P(\chi_{k-N}|Z^{k-N})}{P({\bar{\chi }}_{k-N}|Z^{k-N})}+\Lambda_{k-N}}{\left\{1-{\left(\pi_{11}\right)}^{N}\right\}\frac{P(\chi_{k-N}|Z^{k-N})}{P({\bar{\chi }}_{k-N}|Z^{k-N})}+1}\\ = & \;\left(\Lambda_{k-N}\right)\frac{\left[\frac{1}{1-{\left(\pi_{11}\right)}^{N}}+\frac{P(\chi_{k-N}|Z^{k-1-N})}{P({\bar{\chi }}_{k-N}|Z^{k-1-N})}\right]}{\left[\frac{1}{1-{\left(\pi_{11}\right)}^{N}}+\frac{P(\chi_{k-N}|Z^{k-N})}{P({\bar{\chi }}_{k-N}|Z^{k-N})}\right]} \end{array}$$

Note that the past information of the conventional PTE at time k−N is required to calculate the function $\Psi_{k-N}$ so that the proposed method should be operated with the conventional PTE in parallel.

## References

[1] Alabaster C. (2012). Pulse Doppler Radar: Principles, Technology, Applications.

[2] Richards M.A., Scheer J.A., Holm W.A. (2010). Principles of Modern Radar: Basic Principles.

[3] Nathanson F.E., Reilly J.P., Cohen M.N. (1999). Radar Design Principles: Signal Processing and the Environment.

[4] Magaz B., Belouchrani A., Hamadouche M. (2011). A new adaptive linear combined CFAR detector in presence of interfering targets. Prog. Electromagn. Res..

[5] Hong S.W., Han D.S. (2014). Performance analysis of an environmental adaptive CFAR detector. Math. Probl. Eng..

[6] Liu X., Xu S., Tang S. (2020). CFAR strategy formulation and evaluation based on fox’s h-function in positive alpha-stable sea clutter. Remote Sens..

[7] Mušicki D., Nešić D. Extremum seeking control of ill-defined exponential process. Proceedings of the 2011 Chinese Control and Decision Conference.

[8] Mušicki D., Evans R., Stanković S. (1994). Integrated probabilistic data association. IEEE Trans. Autom. Control.

[9] Challa S., Morelande M.R., Mušicki D., Evans R.J. (2011). Fundamentals of Object Tracking.

[10] Bar-shalom Y., Daum F., Huang J. (2009). The probabilistic data association filter. IEEE Control Syst. Mag..

[11] Bar-Shalom Y., Fortmann T.E. (1988). Tracking and Data Association.

[12] Li X.R., Li N. (2000). Integrated real-time estimation of clutter density for tracking. IEEE Trans. Signal Process..

[13] Li N., Li X.R. (2001). Target perceivability and its applications. IEEE Trans. Signal Process..

[14] Vela G.D.M., Portas J.A.B., Corredera J.R.C. (1998). Probability of false alarm of CA-CFAR detector in Weibull clutter. Electron. Lett..

[15] Bucciarelli T. (1985). CFAR problems in Weibull clutter. Electron. Lett..

[16] Shor M., Levanon N. (1991). Performances of order statistics CFAR. IEEE Trans. Aerosp. Electron. Syst..

[17] Song T.L., Mušicki D. (2011). Adaptive clutter measurement density for improved target tracking. IEEE Trans. Aerosp. Electron. Syst..

[18] Mušicki D., Evans R. (2004). Clutter map information for data association and track initialization. IEEE Trans. Aerosp. Electron. Syst..

[19] Chakravorty R., Mušicki D., Challa S. A fixed lag IPDA smoothing for target tracking in clutter. Proceedings of the 2006 9th International Conference on Information Fusion.

[20] Song T.L., Mušicki D. (2010). Target existence based resource allocation. IEEE Trans. Signal Process..

[21] Fante R.L. (2001). Central limit theorem: Use with caution. IEEE Trans. Aerosp. Electron. Syst..

[22] Choi Y., Chung W.K. (2004). PID Trajectory Tracking Control for Mechanical Systems.

[23] Khalil H.K. (2015). Nonlinear Control.

[24] Billingsley J.B., Farina A., Gini F., Greco M.V., Verrazzani L. (1999). Statistical analyses of measured radar ground clutter data. IEEE Trans. Aerosp. Electron. Syst..

